# Evaluation of Endo 10 mobile application as diagnostic tool in endodontics

**DOI:** 10.4317/jced.60342

**Published:** 2023-08-01

**Authors:** Allan Abuabara, Juliana-Pierdona de Castro, Maria-Eduarda-Nunis Locks, Ana-Paula-Testa Pezzin, Natanael-Henrique-Ribeiro Mattos, Cristiano-Miranda de Araújo, Erika-Calvano Kuchler, Flares Baratto-Filho

**Affiliations:** 1 University of the Region of Joinville (Univille), Joinville, Santa Catarina, Brazil; 2 Tuiuti University of Paraná (UTP), Curitiba, Paraná, Brazil; 3 University of Regensburg, Regensburg, Germany

## Abstract

**Background:**

Endodontic diagnosis can be compared to a puzzle, requiring the interpretation of a series of clinical and imaging data. Mobile health, especially mobile application (apps), can assist professionals in endodontic diagnosis. This work aims to evaluate an app - Endo 10 app, designed to assist pulpal and periapical diagnosis based on the patient’s signs and symptoms and radiographic data.

**Material and Methods:**

A total of 41 dental students and dentists with different levels of expertise (10 multi-specialty clinic professors, 17 residents in endodontics and 14 dental students) were included. The System Usability Scale (SUS) was used to evaluate usability and the Davis’ technology acceptance model was used to evaluate usefulness of Endo 10 app. The Mann-Whitney test was performed to compare SUS scores between professors and undergraduate dental students and to compare questions 6 and 7 of the utility test and verify whether participants who understood that the technology was useful also better understood the concepts of endodontic diagnosis. The agreement between professor’s diagnosis with the app and professor without the app, and between professor and residents in endodontics with the app were evaluated.

**Results:**

The SUS score at the 50th percentile was 77.5, graded as acceptable. No significant difference was observed in the SUS scores when analyzing professors and dental students separately (*p* = 0.442). Usefulness test showed positive responses ranging between 72% - 100%. No statistically significant difference was observed between questions 6 and 7 of the utility test (*p* = 0.206), indicating that the group of participants who understood that the technology was useful in endodontic diagnosis was associated with the agreement that the application helped to better understand the concepts related. The diagnosis agreement between professor in the common diagnosis process and professor with app was 100% (31) of cases. The concordance between professor and residents in endodontics with the app was 71% (22) of cases. The differences were associated with resident’s misinterpreting the patient’s data.

**Conclusions:**

The Endo 10 app reached the usability and usefulness requirements. It proved accurate in diagnosing pulpal and periapical pathologies.

** Key words:**Dental education, endodontics, diagnosis, smartphone, dental informatics.

## Introduction

The digital revolution and growth of the Internet and mobile technology have led to many innovations in the area of electronic learning (1). The healthcare area has followed a global trend towards the digitalization of processes, from the development of health education programs to the implementation of electronic medical records, medical calculators, diagnostic tools and patient monitoring. Mobile equipment that allow quick exchanges of information, such as smartphones, are increasingly important in many areas (2). Mobile health applications (apps) can provide access to evidence-based health information, education, and treatment to end users on a global scale. Currently, there are thousands of mobile health apps (free and paid) publicly available, including apps in the dental-health related (2-4).

In the field of endodontics, the digital revolution has significantly contributed to the enhancement of dental treatment and patient care over the course of decades. As early as 1987, computer simulation programs for endodontic diagnosis were already reported (5). An search on September 4, 2022, in Apple App Store and Google Play Store using the descriptor “endodontics” found an app to assess the difficulty level of an endodontic case (AAE EndoCase app), an app to calculate dose drugs that aids for prescribing medications and also helps with calculating maximum anesthetic dosages (Dental Drugs app), an app that features a tool for measuring canal curvature, tooth inclination and lengths (EndoPrep app), and an app for endodontic diagnosis (Endo 10 app).

**Figure 1:**
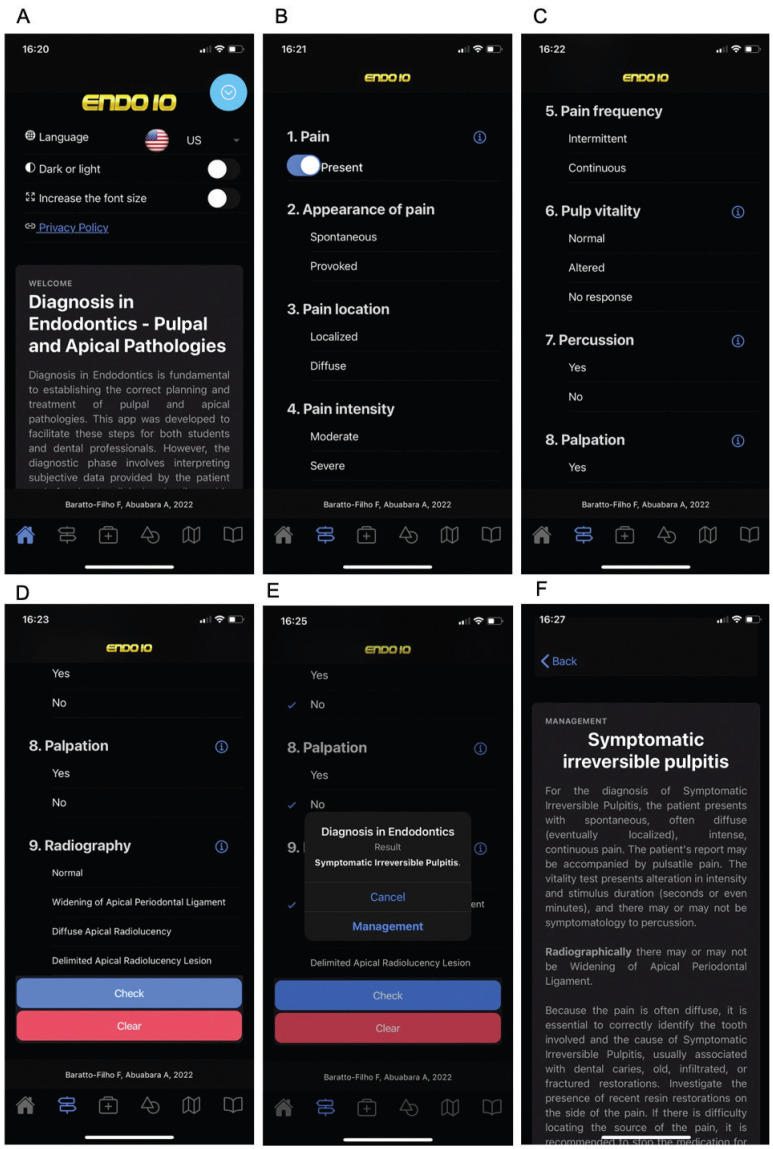
Screens of Endo 10 app: A: Initial, B-C-D: Diagnosis section, E-F: Result and management example.

Endodontic diagnosis requires both clinical and radiographic examinations, including periodontal evaluation and clinical testing (pulp and periapical tests), and is compared to a puzzle (6). In response to this complexity, Endo 10 app was developed to provide endodontists, general dentists and dental students a tool to assist in pulpal and periapical diagnosis and treatment based on the patient’s signs and symptoms and radiographic data. This freely downloaded app (Endo 10, available in English, Spanish, Portuguese) uses a step-by-step process, which recognize the key signs and symptoms in pulpal and periapical diagnosis (7,8). During the diagnosis process it presents a set of 9 questions, including information about the pain (present or absent; if present, appearance, location, intensity and frequency), pulp vitality, periapical tests (percussion and palpation) and periapical radiograph (Fig. 1). The possible results are: Normal Pulp, Reversible Pulpitis, Tendency to Reversible Pulpitis, Symptomatic Irreversible Pulpitis, Tendency to Symptomatic Irreversible Pulpitis, Pulp Necrosis, Acute Apical Pericementitis, Tendency to Acute Apical Pericementitis, Acute Apical Abscess and Symptomatic Apical Periodontitis, Tendency to Acute Apical Abscess, Chronic Apical Abscess and Asymptomatic Apical Periodontitis, Tendency to Chronic Apical Abscess and Asymptomatic Apical Periodontitis, Chronic Reagudized Apical Abscess (Phoenix Abscess) and Symptomatic Apical Periodontitis, Tendency to Chronic Reagudized Apical Abscess (Phoenix Abscess) and Symptomatic Apical Periodontitis, Apical Granuloma and Apical Cyst, Tendency to Apical Granuloma and Apical Cyst, Acute Apical Granuloma and Cyst, Periodontal Abscess, Tendency to Periodontal Abscess, Periapical Cemento-osseous Dysplasia and Inconsistent Information. The results described as “Tendency to” were created to minimize misinterpretation, directing the probable diagnosis. The result “Inconsistent Information” indicates that there is a significant inconsistency among the responses filled out for the 9 items.

App technology can be useful in endodontic diagnosis. The validation of Endo 10 app will aid dental professionals, students and dental educational use. Therefore, the aim of this study was to investigate reliability of the Endo 10 app among undergraduate dental students and dentists with different levels of experience in determining pulpal and periapical diagnosis. The usability, the usefulness and the diagnosis agreement were evaluated.

## Material and Methods

-Study design and ethical considerations

This study was approved by the Ethics Committee of University. The sample size estimation was obtained following previous studies (9-11).

The set were the Dental Clinics at the School of Dentistry at Univille University (Joinville, SC, Brazil), Tuiuti University of Paraná (Curitiba, PR, Brazil) and Dental Institute of the Americas (Curitiba, PR, Brazil). A total of 41 dental students and dentists with different levels of expertise (10 multi-specialty clinic professors, 17 residents in endodontics and 14 undergraduate dental students of the 8th and 10th semesters) were included. The 10 multi-specialty clinic professors represent 10 professors of different clinical specialties, such as restorative dentistry, prosthodontics, surgery, periodontics, endodontics. These professors provide tutoring and academic counseling to students during the patient assistance. This study also included 30 patients (31 teeth) who needed endodontic diagnosis during dental care. Teeth with previous endodontic treatments, root fractures and unrestorable teeth were excluded from the present study.

**Figure 2:**
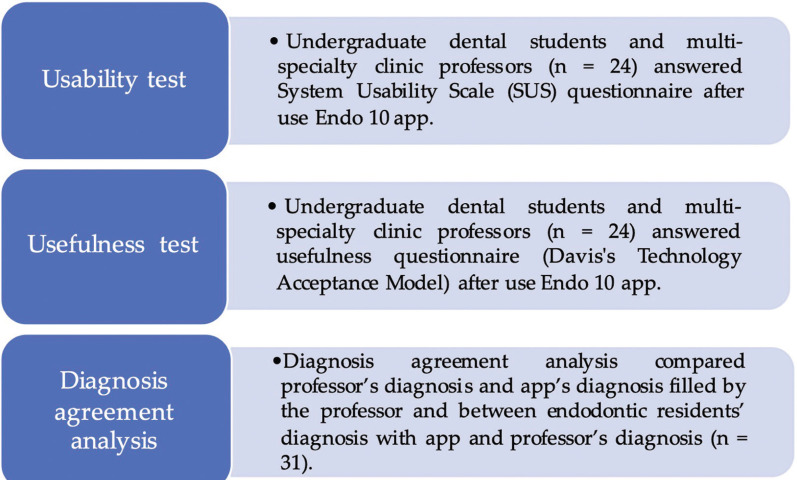
Steps of usability, usefulness and diagnosis agreement tests.

This study evaluated the usability, usefulness and reliability of the Endo 10 app (Fig. 2). The usability test evaluated the application’s ease of use, while the usefulness test evaluated the technology acceptance. The app’s reliability was evaluated by comparing the endodontic diagnosis in the common process and using the app. Each step is described below.

-Tools

The mobile application Endo 10 app (Crescendo Treinamentos Avançados Ltd) was installed on each cellphone, including Android and iOS. The latest version (2.1.8) updated at February 17, 2022 (Apple App Store) and February 16, 2022 (Google Play Store), and Portuguese language was used. After free use of the Endo 10 app, the multi-specialty clinic professors and undergraduate dental students answered the usability test (System Usability Scale - SUS) (Supplement 1) (http://www.medicinaoral.com/medoralfree01/aop/jced_60342_s01.pdf), a valid and reliable measuring tool questionnaire, and the usefulness test based on Davis’s Technology Acceptance Model (Technology Acceptance Model - TAM) (Supplement 1) (http://www.medicinaoral.com/medoralfree01/aop/jced_60342_s02.pdf), both are methods of testing used in the development of health applications (12-17). The tests were available for 30 days. The usefulness questionnaire was adapted to the context of endodontic diagnosis. Demographic data were collected, including age, sex, smartphone operating system and previous experience using mobile applications. The questionnaires were filled out on Google Forms. All data was collected from August to November 2022.

The agreement between professor’s diagnosis (Ph.D. in Endodontics) with the app and professor in the common diagnosis process without the app, and between professor and residents in endodontics with the app were evaluated. The app was filled following the 9 questions proposed by Endo 10 app: pain (present or absent; if present, appearance, location, intensity and frequency), pulp vitality, periapical tests (percussion and palpation) and periapical radiograph.

Pain perception was self-reported. Absence of pain was considered when the patient referred to no pain. Provoked pain was considered when the pain was associated with transient stimuli, such as cold, heat and sweetness, and spontaneous pain is associated when the pain arises without a cause defined. Intermittent pain was considered when the patient referred to the pain to come and go, and continuous pain when it was constant. Localized pain was considered when the patient identified the tooth that caused the pain, while in diffuse pain, the patient was unsure of the pain location. On a scale of zero to ten, moderate was considered when pain up to 5 or 6 and severe above 7. Pulp sensibility (cold thermal test) was performed with double ended ear bud sprayed with 1, 1, 1, tetrafluoroethene (Endo ice refrigerant spray, Coltene/Whaledent Inc., Mahwah, NJ) outside the range of subject’s vision. The bud was held back until it was frosty with the maximum of 7 seconds, following which, it was applied on the middle third of the labial surface of the teeth. Normal vitality tests were associated with discomfort that disappeared immediately after the stimulus was removed. Altered vitality tests were associated with lingering sensitivity that remained for a few seconds or minutes after removal of the stimulus. Negative vitality was observed when the patients did not respond to the thermal test. Positive percussion and palpation were associated, respectively, with sensitivity to a vertical or horizontal percussion using mirror handle and digital palpation. During the periapical radiography examination, it was observed the distinct situations: normal, widening of apical periodontal ligament, diffuse apical radiolucency areas, delimited apical radiolucency areas. Professor and dental residents were always supervised by the responsible researcher. Undergraduate dental students did not participate in this test.

-Statistical analysis

The SUS test is a standardized questionnaire with 10 items, each with five steps anchored with “disagree vehemently” and “agree completely”. It is a mixed-tone questionnaire in which the odd-numbered items have a positive tone, and the even-numbered items have a negative tone (Supplement 1) (http://www.medicinaoral.com/medoralfree01/aop/jced_60342_s01.pdf). The participants ranked each question from 1 to 5 (5 means they “agree completely”, while 1 means they “disagree vehemently”). The first step in scoring a SUS is determining each item’s score contribution. For each of the odd-numbered questions (positively worded questions), it was subtracted 1 from the value. For each of the even-numbered questions (negatively worded questions), it was subtracted from 5. These new values were added up to obtain the total score. Then, to get the overall SUS this value was multiplied by 2.5, converting the original scores of 0-40 to 0-100 (0 is very poor perceived usability and 100 represents excellent perceived usability). The results were evaluated at the 50th percentile and were graded according to Bangor *et al*. (16) and Lewis and Sauro (17) rankings. Values above 68 are classified as accepTable. The Mann-Whitney test was performed to compare SUS scores between professors and undergraduate dental students.

Descriptive statistics was used to describe the usefulness test results and diagnosis agreement results. The Mann-Whitney test was performed to compare questions 6 and 7 of the utility test and verify whether participants who understood that the technology was useful also better understood the concepts of endodontic diagnosis.

Data extraction was performed using Microsoft Excel software. Analyses were performed with Jamovi software v.2.3.17. (Supplement 3,4) (http://www.medicinaoral.com/medoralfree01/aop/jced_60342_s03.pdf), (http://www.medicinaoral.com/medoralfree01/aop/jced_60342_s04.pdf).

## Results

-Demographic data of participants

A total of 10 multi-specialty clinic professors and 14 undergraduate dental students were included in the usability and usefulness tests (n = 24). All participants who underwent the usability and usefulness tests had previous experience using mobile applications, and most (91.67%) already used apps for educational or professional purposes. The mean age of professors and undergraduate dental students was 58 ± 8 and 25 ± 6 years, respectively. The mean age of the combined group of professors and dental students was 39 years ± 17. The female participants were 15 (62.5%) and male 9 (37.5%). In Table 1 it is possible to observe demographic data of participants.

**Table 1:**
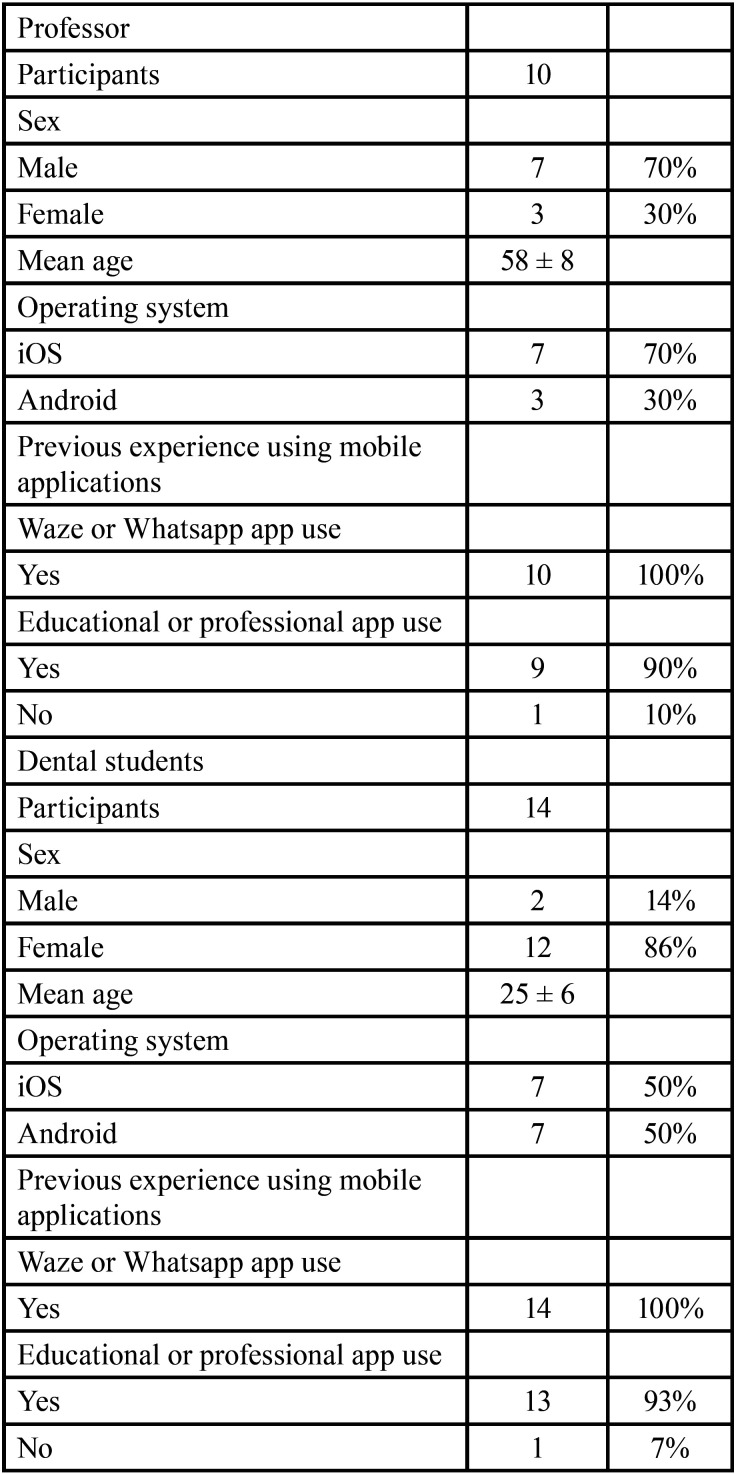
Demographic data of participants who underwent the usability and usefulness tests.

-System Usability Scale test results

The SUS score at the 50th percentile was 77.5 (Table 2). The Mann-Whitney test did not show a significant difference in the SUS score when analyzing professors and dental students separately (*p* = 0.442).

**Table 2:**
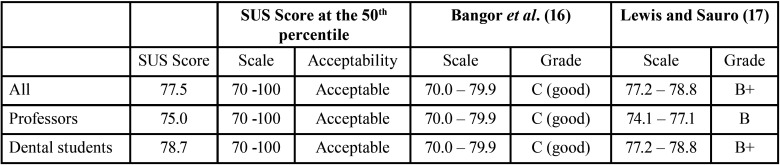
System Usability Scale test results.

-Usefulness test results

In Table 3 it is possible to observe the answers to the questions of the usefulness assessment questionnaire. Grouping positive responses (I strongly agree and I agree, It helps a lot and It Helps), the values ranged from 96% - 100%, except for question 7, “It has helped me to better understand the concepts related to endodontic diagnosis and treatment” (72%). No statistically significant difference, evaluated by the Mann-Whitney test, was observed between questions 6 and 7 (*p* = 0.206), indicating that the group of participants who understood that the technology was useful in endodontic diagnosis was associated with the agreement that the application helped to better understand the concepts related.

**Table 3:**
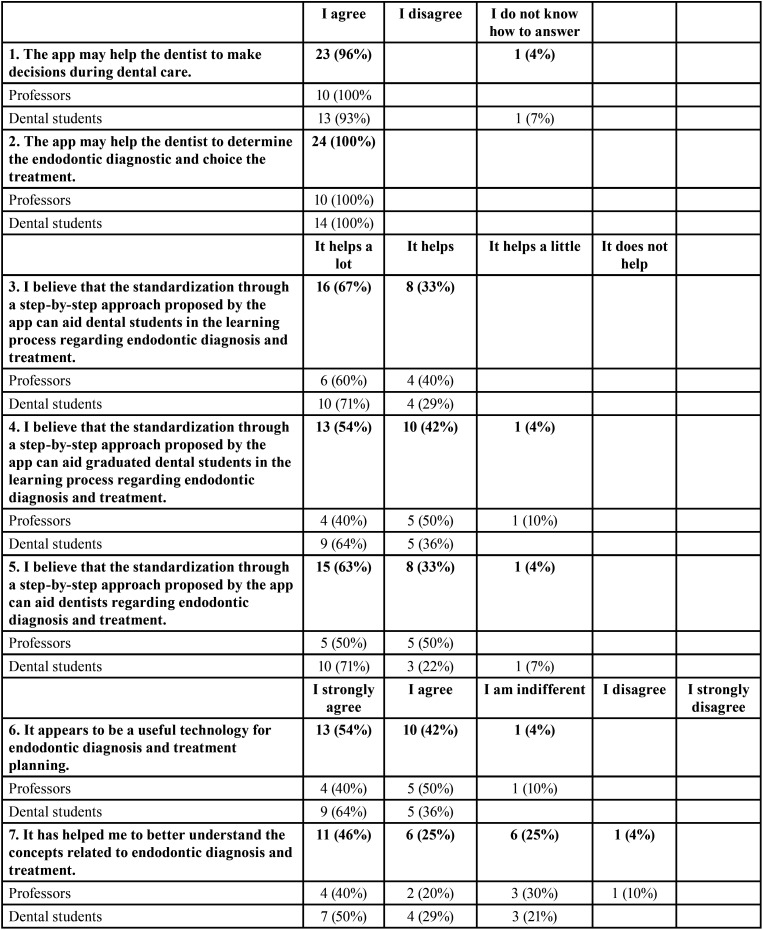
Data expressed as absolute frequency and percentage for answers to the usefulness evaluation questionnaire.

-Diagnosis agreement results

The diagnosis agreement between professor in the common diagnosis process and professor diagnosing with the app was 100% (31) of cases. The concordance between professor and residents in endodontics with the app was 71% (22) of cases (Table 4). The differences were associated with resident’s misinterpreting the patient’s data in 9 cases: 5 in the radiographic examination, 2 in the vitality test, 1 in the clinical examination (palpation), and 1 vitality test, radiographic examination and characteristics of pain. When the app was filled in with the clinical and radiographic data interpreted by the professor, the diagnosis agreement was 100% (31 of 31).

**Table 4:**
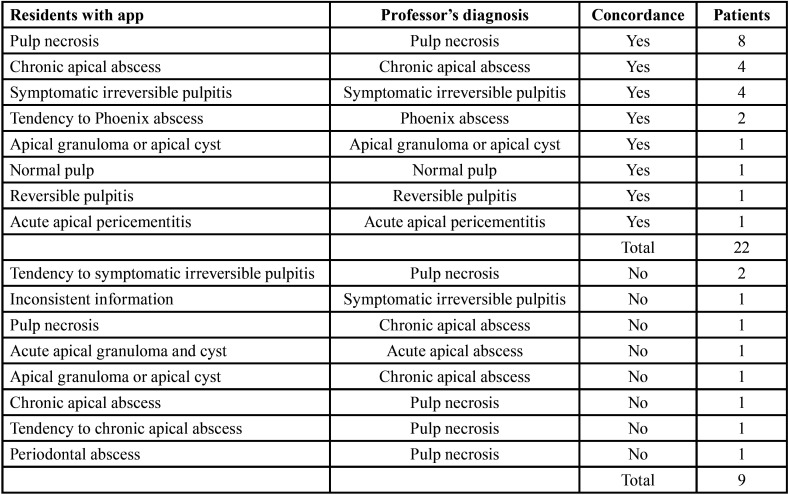
Diagnosis agreement.

## Discussion

The development of mobile applications in the healthcare field has been showing an exponential increase (9,18-20). This trend is also observed in the dental area (4,21,22). During the design of this study, from January to August 2022, only Endo 10 was found as an app related to endodontic diagnostic assistance in Apple App Store and Google Play Store. The app was based on current evidence and experts’ knowledge and experience. Based on a probability tree of nine clinical and radiological data, the app tests these combinations and suggests the diagnosis. Therefore, in the present study we performed an initial analysis of the Endo 10, which can be used in clinical and teaching environments.

Different strategies of teaching might present greater efficacy in improvement health sciences’ students and professionals’ knowledge (23). Nowadays, educators must be prepared to respond creatively to these changes associating traditional classroom and mobile devices (23). For example, the use of a mobile app containing reference images can improve the students’ ability to diagnose endodontic complications, suggesting it would be a valuable supplementary tool in dental education (24). Duruk and Gümüşboğa (21) also showed that apps can be an effective training tool to increase the knowledge level of individuals about the emergency management of traumatic dental injuries. In our study, Endo 10 app was also tested in schools of dentistry and undergraduate dental students and multi-specialty clinic professors greatly appreciated the app, recognizing that the app can aid dental students in the learning process regarding endodontic diagnosis and treatment.

One important question for mobile health app is “What makes a good health app?”. There is no robust scorecard to understand and evaluate the different dimensions of mobile health apps (25). The validation, in general, is specific to a single app from a field, and its design is focused on evaluating its usability aspects (25). There are increasing numbers of clinical scoring systems that can include calculations, such as determining the endodontic diagnosis, the difficulty of an endodontic case, calculating dose drugs and measuring the root canal length. These apps can provide information rapidly, but their reliability must be verified to reduce the risk of error. Bierbrier and Wu (26) evaluated the accuracy of smartphone-based medical calculation apps and found that most medical calculating apps provide accurate and reliable results. However, some errors were noted in some functions of some apps. Out of the 1,240 tests conducted the authors found 17 errors (26). Bierbrier and Wu (26) highlight the need for verifying medical apps before use in patient care. In our study, we observed that of the 31 endodontic cases, the diagnosis agreement between professor and professor with app was 100% using Endo 10 app, showing that the app is reliable.

The design of this study followed Nogueira *et al*. (9) and it was adapted to the dental context. The innovation of the present study consisted of evaluating the diagnosis agreement, usability and usefulness of potential users of the Endo 10 app. It is noteworthy that 100% of professors and 93% of students confirmed that the app might help the dentist make decisions during dental care, and 100% confirmed that it might help the dentist determine the endodontic diagnostic and treatment choice. In the usefulness test, the worst result was for question 7, “It has helped me to better understand the concepts related to endodontic diagnosis and treatment”, which got 72% of positive responses. In comparison, for the other questions, this percentage went from 95%. A standardization through a step-by-step approach proposed by the app can aid dental students in learning and offer professionals a second opinion tool. The participants who understood that the technology was useful in endodontic diagnosis also agreed that the application helped to better understand the concepts related.

The SUS tool was initially developed to evaluate the usability of engineering and electronic office systems. Nowadays, it is used to evaluate many products and services, such as software, webpages, or mobile apps. It focuses on measuring usability using a Likert scale of 10 elements (25). The SUS is the usability questionnaire that has been cited in more than 1,200 publications, becoming one of the most widely used usability questionnaires (27). Sousa and Dunn Lopez (27) believe that SUS is the strongest of the currently available questionnaires to measure the usability of e-health tools. An advantage for use SUS is that with a small sample, 8-12 users, it is possibly get a good assessment of how people see your system or product (10,11). The SUS score at the 50th percentile of the Endo 10 app was 77.5, with no statistical difference between groups. Hyzy *et al*. (28) concluded that the SUS and the widely accepted benchmark of a mean SUS score of 68 are suiTable for evaluating the usability of digital health apps (28). It needs to look at its percentile ranking to understand how the product or app compares to others. A score above 68, as found in this study, at or around the 50th percentile, means that your app would be considered above average. Bangor *et al*. (16) assigned the adjectives for SUS score: “worst imaginable” (0-25), “poor” (26-39), “acceptable” (40-52), “good” (53-74), “excellent” (75-85), “best imaginable” (86-100). The classification proposed by Lewis and Sauro (17) is carried out by letters as follows: F (0-51.6), D (51.7-62.6), C- (62.7-64.9), C ( 65.0-71.0), C+ (71.1-72.5), B- (72.6-74.0), B (74.1-77.1), B+ (77.2-78.8), A- (78.9-80.7), A (80.8-84.0) and A+ (84.1-100). In Table 2, it is possible to observe SUS test results for the Endo 10 app. According to Bangor *et al*. (16) and Lewis and Sauro (17), the Endo 10 app was rated as C (good) and B+, respectively.

The diagnostic agreement analysis evidences the importance of the interpretation of the subjective data of the patient. Although technology tools such as mobile apps can help the dentist, the interpretation of sign and symptoms highly important. When the Endo 10 app was filled in with the clinical and radiographic data interpreted by the professor (Ph.D.), the gold standard in this study, the diagnosis agreement was 100%. Misinterpretation of patient data by residents was found in 29% (9) of cases. The nine patients that presented diagnostic disagreement between the endodontic residents with the app and the professor were analyzed in more detail: in five cases we observed a mistake during the analysis of the periapical radiography, two failed the vitality test conduction, one failed in the periapical test (palpation), and one during the pain characteristics analysis, radiographic examination and vitality test.

It is important to point the few limitations of this study. Regarding the application of the usability and usefulness tests, the bias of the authors knowing part of the participants is noteworthy, which may have inhibited the critical posture of some participants. In addition, two authors are the Endo 10 app’s developers. Therefore, other authors were invited to be part of this study. The Endo 10 app version 2.1.8 does not follow the American Association of Endodontists diagnostic classification and terminology (29). An update should be regarded.

## Conclusions

Endo 10 app has proved to be helpful in pulpal and periapical diagnosis and reached the usability and usefulness requirements. Additional multicenter research and accuracy study are warranted.
